# Changing trends in epidemiology and antifungal susceptibility patterns of six bloodstream *Candida* species isolates over a 12-year period in Kuwait

**DOI:** 10.1371/journal.pone.0216250

**Published:** 2019-05-01

**Authors:** Ziauddin Khan, Suhail Ahmad, Noura Al-Sweih, Eiman Mokaddas, Khalifa Al-Banwan, Wadha Alfouzan, Inaam Al-Obaid, Khaled Al-Obaid, Mohammad Asadzadeh, Ahlam Jeragh, Leena Joseph, Soumya Varghese, Sandhya Vayalil, Omar Al-Musallam

**Affiliations:** 1 Department of Microbiology, Faculty of Medicine, Kuwait University, Safat, Kuwait; 2 Department of Microbiology, Maternity Hospital, Shuwaikh, Kuwait; 3 Department of Microbiology, Ibn-Sina Hospital, Shuwaikh, Kuwait; 4 Department of Microbiology, Al-Amiri Hospital, Kuwait City, Kuwait; 5 Department of Microbiology, Farwaniya Hospital, Farwaniya, Kuwait; 6 Department of Microbiology, Al-Sabah Hospital, Shuwaikh, Kuwait; 7 Department of Microbiology, Mubarak Al-Kabeer Hospital, Jabriya, Kuwait; 8 Department of Microbiology, Al-Adan Hospital, Hadyia, Kuwait; 9 Mycology Reference Laboratory, Department of Microbiology, Faculty of Medicine, Kuwait University, Safat, Kuwait; Lebanese American University, LEBANON

## Abstract

Changing trends in incidence and antifungal susceptibility patterns of six *Candida* species causing candidemia in Kuwait between 2006–2017 are reported. A total of 2075 isolates obtained from 1448 patients were analyzed. Identity of *Candida* species isolates was determined by phenotypic methods and confirmed by PCR amplification/PCR-sequencing of rDNA and/or MALDI-TOF MS. Antifungal susceptibility was determined by Etest. *C*. *albicans* accounted for 539 (37.22%) cases followed by *C*. *parapsilosis* (n = 502, 34.67%), *C*. *tropicalis* (n = 210, 14.5%), *C*. *glabrata* (n = 148, 10.22%), *C*. *krusei* (n = 27, 1.81%) and *C*. *dubliniensis* (n = 22, 1.5%). The comparative percent distribution of *Candida* species causing candidemia between 2006–2011 and 2012–2017 was as follows: *C*. *albicans* 41.8% and 33.1%, *C*. *parapsilosis* complex 32.01% and 37.04%, *C*. *tropicalis* 13.59% and 15.31%, and *C*. *glabrata* 8.77% and 11.51%, *C*. *krusei* 2.0% and 1.7%, and *C*. *dubliniensis* 1.75 and 1.3%, respectively. Three of 371 *C*. *albicans* isolates during 2006–2011 and five of 363 during 2012–2017 were resistant to fluconazole. Among *C*. *parapsilosis* isolates, one of 310 during 2006–2011 and 21 of 446 during 2012–2017 were resistant to this drug. Furthermore, at an epidemiologic cutoff value (ECV) of ≤0.5 μg/ml, 70.1% *C*. *albicans* isolates were wild-type for fluconazole during 2006–2011 as compared to 58.1% during 2012–2017. Likewise, at an ECV of ≤2 μg/ml, 98.0% of *C*. *parapsilosis* isolates were wild-type during 2006–2011 as compared to 93.4% during 2012–2017. Clonal spread of fluconazole-resistant *C*. *parapsilosis* in one major hospital was documented. An 8.8% shift in favor of non-*albicans Candida* species with concomitant increase in MICs between the two periods preludes emergence of fluconazole-resistant candidemia cases in Kuwait.

## Introduction

*Candida* species are a major cause of healthcare-associated bloodstream infection (BSI) worldwide [1, 2]. They are associated with considerable infection-related morbidity and mortality, particularly in intensive care unit (ICU), where at least 50% episodes of candidemia occur [3]. While *C*. *albicans* continues to be the most frequently isolated bloodstream pathogen in most studies, there is a gradual shift towards non-*albicans Candida* species (*C*. *glabrata*, *C*. *tropicalis*, and *C*. *parapsilosis*), which together account for >50% cases of candidemia [1, 2, 4]. Considerable differences exist in the number of cases caused by individual non-*albicans Candida* species depending upon the geographic region, patient population, age and prior exposure to antifungal agents [1, 5]. Regardless of the geographical area, *C*. *albicans* is more frequent in patients aged up to 18 years, the frequency of *C*. *parapsilosis* decreases with age and *C*. *glabrata* is more common among the elderly [1]. Studies from Northern Europe and the USA [4, 6] have reported relatively a higher number of cases due to *C*. *glabrata* as compared to Spain and Brazil, where a higher number of cases were caused by *C*. *parapsilosis* [1]. On the contrary, *C*. *tropicalis* is the most common non-*albicans Candida* species in Asia [7]. Like many other countries, candidemia is also an important cause of BSI both in adult and pediatric patients in Kuwait [8–15]. In a previous study, we have reported species distribution and antifungal susceptibility profile of bloodstream isolates obtained during a 10-year period (1996–2005) [8]. Herein, we aimed to identify epidemiological changes that have occurred in the distribution and antifungal susceptibility pattern of six *Candida* species during a 12-year period since 2006.

## Materials and methods

### Bloodstream yeast isolates and phenotypic methods for identification

*Candida* species isolates were grown from blood collected after obtaining informed verbal consent from patients for routine diagnosis. The isolates were referred to Mycology Reference Laboratory (MRL) as a part of routine patient care for identification and susceptibility testing. Since this retrospective study did not involve human participants, no specific ethical approval was required. Patients details were fully anonymized and results on de-identified samples are presented in this manuscript. Six *Candida* species/species complexes viz. *C*. *albicans*, *C*. *parapsilosis* sensu lato, *C*. *tropicalis*, *C*. *glabrata*, *C*. *dubliniensis* and *C*. *krusei* were included. A total of 1448 culture confirmed candidemia cases due to six *Candida* species were recorded during the 12-year study period (2006 to 2017) across Kuwait. Repeat bloodstream isolates were also obtained from several patients with persistent candidemia. The bloodstream isolates were divided into two 6-year study periods that is 2006 to 2011 and 2012 to 2017 to compare relative shift in the prevalence of *Candida* species. The isolates were identified by phenotypic methods which included germ tube test, colony characteristics on CHROMagar Candida and VITEK2 yeast identification system.

### Molecular identification

The identity of the isolates which showed unusual phenotypic and/or antifungal susceptibility pattern or resistance to antifungal drugs was confirmed by PCR amplification/PCR-sequencing of rDNA, performed as described in detail previously [16,17]. *C*. *parapsilosis* sensu stricto were differentiated from *C*. *orthopsilosis* and *C*. *metapsilosis* by a multiplex PCR, as described previously [18, 19]. The identity of the isolates was also confirmed by matrix-assisted laser desorption/ionization time-of-flight mass spectrometry (MALDI-TOF MS; bioMérieux, Marcy l’Etoile, France) as described previously [20].

### Antifungal susceptibility testing

The *in vitro* activity of the antifungal agents was determined by E-test (bioMérieux, Marcy l’Etoile, France). The test was performed in accordance with the manufacturer's instructions. Petri dishes (150 mm diameter) containing 60 ml RPMI agar supplemented with 2% glucose and buffered to pH 7.0 with MOPS were used. The inoculum was applied with cotton swabs using growth suspension prepared in 0.85% NaCl with turbidity adjusted to 0.5 McFarland standard. Plates were incubated at 35°C and read after 24 h. Reference strains of *C*. *albicans* (ATCC 90028) and *Candida parapsilosis* (ATCC 22019) were used for quality control. Susceptibility breakpoints used for interpretation of susceptible, intermediate/ susceptible dose-dependent and resistant strains were those recommended by the Clinical and Laboratory Standards Institute [21]. Due to lack of defined breakpoints for amphotericin B, isolates showing an MIC of ≤1.0/μg ml were taken as susceptible and those with MIC >1μg/ml as resistant [22]. The CLSI epidemiologic cutoff values (ECV) of fluconazole for *C*. *albicans*, *C*. *parapsilosis* and *C*. *tropicalis* were ≤0.5 μg/ml, ≤2 μg/ml and ≤2 μg/ml, respectively, as also proposed by Pfaller & Diekema [23].

### Statistical analysis

Statistical analysis was performed by using chi-square test or Fisher’s exact test as appropriate and probability levels <0.05 by the two-tailed test were considered as statistically significant. Statistical analyses were performed by using WinPepi software ver. 11.65 (PEPI for Windows, Microsoft Inc., Redmond, WA, USA).

## Results

The species distribution of 2075 *Candida* species isolates obtained from 1448 candidemia patients during the 12-year study period are presented in Table 1. *C*. *albicans* was the most frequently isolated species accounting for 539 (37.22%) cases closely followed by 502 (34.67%) cases due to *C*. *parapsilosis* complex isolates including *C*. *orthopsilosis* (n = 5) and *C*. *metapsilosis* (n = 2). The remaining cases were due to *C*. *tropicalis* (n = 210, 14.5%), *C*. *glabrata* (n = 148, 10.22%), *C*. *krusei* (n = 27, 1.81%) and *C*. *dubliniensis* (n = 22, 1.5%) (Table 1). The comparative percent distribution of *Candida* species causing candidemia between 2006–2011 and 2012–2017 was as follows: *C*. *albicans* 41.8% and 33.1%, *C*. *parapsilosis* complex 32.01% and 37.04%, *C*. *tropicalis* 13.59% and 15.31%, and *C*. *glabrata* 8.77% and 11.51%, and *C*. *krusei* 2.0% and 1.7%, respectively (Table 1). There was an overall increase of 8.8% candidemia cases caused by non-*albicans Candida* species during 2012–2017.

**Table 1:** Distribution of 2075 *Candida* species isolated from 1448 candidemia patients obtained during 2006–2011 and 2012–2017.

Species/ complexes	Distribution of candidemia cases and isolates during				Total cases (%)
	2006–2011		2012–2017		
	Cases (%)	Isolates (%)	Cases (%)	Isolates (%)	
*C*. *albicans*	286 (41.8)	405 (41.75)	253 (33.11)	363 (32.85)	539 (37.22)
*C*. *dubliniensis*	12 (1.75)	16 (1.64)	10 (1.30)	11 (0.99)	22 (1.5)
C. parapsilosis*	219 (32.01)	332 (34.22)	283 (37.04)	450 (40.72)	502 (34.66)
*C*. *tropicalis*	93 (13.59)	126 (12.98)	117 (15.31)	150 (13.57)	210 (14.5)
*C*. *glabrata*	60 (8.77)	76 (7.83)	88 (11.51)	115 (10.40)	148 (10.2)
*C*. *krusei*	14 (2.04)	15 (1.54)	13 (1.70)	16 (1.44)	27 (1.8)
**Total**	**684**	**970**	**764**	**1105**	**1448**

*Includes 7 *C*. *orthopsilosis* isolates from 5 cases and 2 *C*. *metapsilosis* isolates from 2 cases

The year-wise distribution of *Candida* species isolates during the study period and the annual incidence rate of candidemia are presented in Table 2. Although *C*. *albicans* was the most frequently isolated species during 2006 to 2012, it was replaced by *C*. *parapsilosis* sensu stricto in the next four years (2013 to 2016) as the most frequently isolated *Candida* species from candidemia patients (numbers shown in bold). The isolation frequency of *C*. *albicans* varied from a low of ~29% in 2013 and 2016 to a high value of 52.2% in 2011, however, it showed a general declining trend over the study period. On the other hand, the isolation frequency of *C*. *parapsilosis* showed a declining trend during 2006 to 2011 and then an upward trend during 2012 to 2016 (Table 2). *C*. *tropicalis* and *C*. *glabrata* were recorded as the third and fourth most frequently isolated *Candida* species in Kuwait, respectively, and the isolation frequency of both the species generally showed an upward trend during the study period (Table 2). Consistent with these data, the ratio of isolation of *C*. *albicans* versus all six *Candida* species isolates declined from 0.46 in 2006 to 0.33 in 2017. The annual incidence rate (number of candidemia cases per 100, 000 population) showed a declining trend (from 4.45 to 3.5) in Kuwait over the study period (Table 2). One-third of candidemia cases (n = 480) occurred among neonates and there was a >2-fold increase in isolation of *C*. *parapsilosis* from neonates in Maternity Hospital during 2012–2017 (n = 115) as compared to 2006–2011 (n = 49). *C*. *glabrata* was isolated in greater number from elderly immunocompromised patients including those with cancer, that is, 23 isolates in 2012–2017 as compared to only 4 in 2006–2011. None of the bloodstream *C*. *glabrata* isolate was identified as *C*. *nivariensis* or *C*. *bracarensis* by PCR amplification and/or PCR-sequencing of rDNA.

**Table 2:** Year-wise distribution of *Candida* species and population-based incidence of candidemia in Kuwait (2006–2017).

Year	*C*. a*lbicans*	*C*. *parapsilosis*	*C*. *tropicalis*	*C*. *glabrata*	*C*. *dubliniensis*	*C*. *krusei*	Total	Ratio of *C*. *albicans*/ all *Candida* species	Total Kuwait population	Annual incidence/10^5^ population*
2006	**49**	41	9	4	2	1	106	0.46	2377258	4.45
2007	**44**	39	12	8	0	1	104	0.42	2503410	4.15
2008	**43**	35	21	12	0	5	116	0.37	2652340	4.37
2009	**43**	36	17	14	8	0	118	0.36	2818939	4.18
2010	**49**	45	18	11	2	4	129	0.38	2998083	4.3
2011	**58**	23	16	11	0	3	111	0.52	3191051	3.47
2012	**41**	26	16	15	1	0	99	0.41	3396556	2.91
2013	29	**37**	16	15	0	2	99	0.29	3598386	2.75
2014	46	**63**	17	16	3	2	147	0.31	3782450	3.88
2015	50	**55**	19	12	2	4	142	0.35	3935794	3.6
2016	39	**58**	20	13	0	2	132	0.29	4052584	3.25
2017	**48**	44	29	17	4	3	145	0.33	4136528	3.5

*Mean annual incidence is 3.69/10^5^ population.

Numbers in bold indicate most common species in that year.

The antifungal susceptibility profiles of *Candida* species isolates obtained during the study period were determined by Etest against four antifungal drugs, viz. fluconazole, voriconazole, amphotericin B and caspofungin and the data are presented in Tables 3 and 4. None of the *C*. *albicans* (n = 741), *C*. *parapsilosis* sensu lato (n = 763), *C*. *tropicalis* (n = 264), and *C*. *dubliniensis* (n = 26) isolates tested in this study was resistant to amphotericin B as their MIC values were ≤1.0/μgml. Only one of 31 *C*. *krusei* and 2 of 190 *C*. *glabrata* isolates were resistant to amphotericin B as their MIC values were >1.0 μgml. On the other hand, resistance to fluconazole and cross-resistance to voriconazole was detected more frequently. Only eight *C*. *albicans* isolates were resistant to fluconazole and five of these were isolated during 2012–2017. Three of these isolates also showed cross-resistance to voriconazole. Although resistance to fluconazole in *C*. *albicans* increased during 2012 to 2017 compared to 2006 to 2011, it was not statistically significant (*P* = 0.501). However, resistance rate among *C*. *parapsilosis* sensu stricto isolates increased over time for both fluconazole and voriconazole and these differences were statistically significant (*P* = 0.000 and *P* = 0.021, respectively) (Table 3). Resistance to fluconazole was also detected more frequently among *C*. *glabrata* isolates during 2012 to 2017 compared to 2006 to 201, however, the difference was not statistically significant (*P* = 0.070) (Table 3).

**Table 3:** Prevalence of antifungal drug resistance among bloodstream isolates of major *Candida* species/complexes tested during 2006–2011 and 2012 to 2017 in Kuwait^a^.

Antifungal drug	Duration	*Candida albicans*		*Candida parapsilosis*		*Candida orthopsilosis*		*Candida tropicalis*		*Candida glabrata*		*Candida krusei*	
		No. tested	Resistant (%)	No. tested	Resistant (%)	No. tested	Resistant (%)	No. tested	Resistant (%)	No. tested	Resistant (%)	No. tested	Resistant (%)
Amphotericin B	2006–2011	378	0	310	0	5	0	114	0	75	1 (1.4)	15	1 (6.7)
	2012–2017	363	0	446	0	2	0	150	0	115	1 (1.1)	16	0
Fluconazole	2006–2011	371	3 (0.8)	310	1 (0.3)	5	0	112	1 (0.8)	74	3 (4.1)	13	0
	2012–2017	363	5 (1.4)	446	**21 (4.7)**^b^	2	0	150	1 (0.7)	115	14 (12.2)	16	0
Voriconazole	2006–2011	331	4 (1.2)	274	0	5	0	108	1 (0.9)	62	0	14	0
	2012–2017	304	4 (1.3)	360	**7 (1.9)**^b^	1	0	117	0	84	0	12	0

^a^Bloodstream isolates of all *Candida* species tested in this study were susceptible to caspofungin and all isolates of *C*. *dubliniensis* were additionally susceptible to amphotericin B, fluconazole and voriconazole

^b^Significantly higher rates of resistance to fluconazole and voriconazole observed for *C*. *parapsilosis* during 2012 to 2017 compared to 2006 to 2011 period are highlighted in boldface numbers

**Table 4:** Comparison of antifungal susceptibility of bloodstream isolates of five major *Candida* species/complexes tested during 2006–2011 and 2012–2017.

*Candida* Species/Antifungal agent	2006–2011						2012–2017					
	No. of isolates	Range (μg/ml)	MIC50	MIC90	GM (μg/ml)	% Resistance (n)**	No. of isolates	Range (μg/ml)	MIC50	MIC90	GM (μg/ml)	% Resistance (n)**
**Amphotericin B**												
*C*. *albicans*	378	0.002–0.75	0.047	0.125	0.04 ± 0.07	0	363	0.002–0.38	0.047	0.125	0.04 ± 0.05	0
*C*. dubliniensis*	15	0.002–0.125	0.012	0.047	0.011 ± 0.03	0	11	0.003–0.094	0.008	0.032	0.013 ± 0.025	0
*C*. *parapsilosis*	310	0.002–1	0.023	0.094	0.02 ± 0.11	0	446	0.002–0.75	0.023	0.094	0.02 ± 0.08	0
*C*. orthopsilosis*	5	0.008–0.125	0.023	0.125	0.02 ± 0.04	0	2	0.008–0.064	-	-	0.022 ± 0.039	0
*C*. *tropicalis*	114	0.002–0.75	0.047	0.19	0.04 ± 0.11	0	150	0.003–0.5	0.064	0.19	0.06 ± 0.07	0
*C*. *glabrata*	75	0.004–2	0.094	0.25	0.08 ± 0.24	1.4 (1)	115	0.002–32	0.125	0.38	0.10 ± 2.97	1.07 (1)
*C*. *krusei*	15	0.094–6	0.125	1	0.14 ± 1.51	6.66 (1)	16	0.012–0.5	0.19	0.25	0.13 ± 0.14	0
**Fluconazole**												
*C*. *albicans*	371	0.004- ≥256	0.38	1	0.39 ± 22.91	0.8 (3)	363	0.094–24	0.5	1	0.55 ± 1.86	1.37 (5)
*C*. dubliniensis*	15	0.016–0.75	0.25	0.75	0.24 ± 0.22	0	11	0.125–1	0.38	0.75	0.34 ± 0.26	0
*C*. *parapsilosis*	310	0.016–32	0.38	1	0.33 ± 1.88	0.32 (1)	446	0.047-≥256	0.75	1.5	0.69 ± 41.24	4.7 (21)
*C*. orthopsilosis*	5	0.19–0.5	0.25	0.5	0.25 ± 0.15	0	2	0.25–0.38	-	-	0.308 ± 0.091	0
*C*. *tropicalis*	112	0.012–8	0.75	2	0.55 ± 1.05	0.8 (1)	150	0.023-≥256	0.38	1	0.43 ± 20.86	0.66(1)
*C*. *glabrata*	74	0.064-≥256	0.094	32	7.17 ± 49.68	4.05 (3)	115	0.75-≥256	12	64	12.43 ± 54.83	12.17 (14)
*C*. *krusei*	13	6–64	24	64	21.65 ± 21.57	-	16	0.38–48	16	32	14.11 ± 13.25	-
**Voriconazole**												
*C*. *albicans*	331	0.002-≥32	0.023	0.094	0.02 ± 3.03	1.2 (4)	304	0.002–1.5	0.023	0.125	0.02 ± 0.16	1.3 (4)
*C*. dubliniensis*	15	0.004–0.19	0.016	0.064	0.02 ± 0.04	0	11	0.006–0.094	0.008	0.023	0.011 ± 0.025	0
*C*. *parapsilosis*	274	0.002–0.38	0.016	0.047	0.015 ± 0.037	0	360	0.002-≥32	0.023	0.094	0.024 ± 2.39	1.94 (7)
*C*. orthopsilosis*	5	0.006–0.125	0.008	0.125	0.014 ± 0.05	0	1	0.012	-	-	-	0
*C*. *tropicalis*	108	0.003–2	0.047	0.125	0.046 ± 0.196	0.92 (1)	117	0.004–0.75	0.047	0.125	0.042 ± 0.079	0
*C*. *glabrata*	62	0.016–4	0.125	0.75	0.15 ± 0.58	-	84	0.032-≥32	0.25	1.5	0.31 ± 3.51	-
*C*. *krusei*	14	0.047–0.5	0.094	0.38	0.13 ± 0.14	0	12	0.047–0.38	0.125	0.38	0.16 ± 0.12	0
**Caspofungin**												
*C*. *albicans*	65	0.002–0.38	0.094	0.19	0.05 ± 0.07	0	363	0.002–0.38	0.094	0.19	0.08 ± 0.06	0
*C*. dubliniensis*	1	0.047	-	-	-	0	11	0.032–0.19	0.064	0.19	0.08 ± 0.06	0
*C*. *parapsilosis*	32	0.012–1.5	0.38	1	0.27 ± 0.34	0	446	0.002–1	0.38	0.5	0.29 ± 0.15	0
*C*. orthopsilosis*	1	0.38	-	-	-	0	2	0.38–0.5	-	-	0.43 ± 0.084	0
*C*. *tropicalis*	29	0.008–0.75	0.094	0.25	0.093 ± 0.147	0	150	0.002–0.38	0.125	0.19	0.10 ± 0.07	0
*C*. *glabrata*	9	0.047–0.38	0.125	0.25	0.129 ± 0.107	0	115	0.002–0.38	0.125	0.19	0.11 ± 0.07	0
*C*. *krusei*	5	0.125–0.38	0.38	0.38	0.26 ± 0.12	0	16	0.19–0.5	0.25	0.38	0.26 ± 0.104	0

*CLSI breakpoints as recommended for *C*. *albicans* and *C*. *parapsilosis* [19].

Fluconazole: *C*. *albicans*, *C*. *parapsilosis*, *C*. *tropicalis*: ≤ 2 μg/ml (S), 4 μg/ml (SDD), ≥ 8 μg/ml (R); *C*. *glabrata*: ≤ 32 μg/ml (SDD), ≥ 64 μg/ml (R)

Voriconazole: *C*. *albicans*, *C*. *parapsilosis*, *C*. *tropicalis*: ≤ 0.12 μg/ml (S), 0.25–0.5 μg/ml (I), ≥1 μg/ml; *C*. *krusei*: ≤ 0.5 μg/ml (S), 1 μg/ml (I), ≥ 2 μg/ml (R)

**Figures in parentheses indicate number of patients yielding resistant isolates.

The MIC_50_, MIC_90_ and the geometric mean of the MICs for amphotericin B and caspofungin remained nearly same for different *Candida* species during the study period (Table 4). None of the bloodstream isolates of *C*. *albicans*, *C*. *parapsilosis*, *C*. *tropicalis* and *C*. *dubliniensis* were resistant to amphotericin B and geometric mean of the MICs remained stable between the two study periods (Table 4). Only two isolates of *C*. *glabrata* were resistant to amphotericin B (MIC ≥1μg/ml), one each among the 75 isolates tested during 2006–2011 and 115 isolates during 2012–2017. Three of 371 (0.8%) *C*. *albicans* isolates tested during 2006–2011 and 5 of 363 (1.37%) isolates during 2012–2017 were resistant to fluconazole (MIC ≥8 μg /ml) (Tables 3 and 4). Three of these isolates also showed cross-resistance to voriconazole.

The comparative distribution of MIC values for fluconazole for four major *Candida* species (viz. *C*. *albicans*, *C*. *parapsilosis*, *C*. *tropicalis* and *C*. *glabrata*) isolates during the study period is presented in Table 5. The *C*. *albicans* and *C*. *parapsilosis* isolates showed an increasing trend in MICs to fluconazole. At an ECV of ≤0.5 μg/ml, 260 of 371 (70%) *C*. *albicans* isolates tested during 2006–2011 were wild-type for fluconazole as against 211 of 363 (58.1%) tested during 2012–2017 (Table 5). Emergence of fluconazole resistance was more pronounced among bloodstream isolates of *C*. *parapsilosis*. Of 310 *C*. *parapsilosis* isolates, only one isolate (0.3%) was resistant to this drug during 2006–2011 as against 21 of 446 (4.7%) isolates during 2012–2017. This observation was also supported by distribution of MIC values of the isolates obtained during the two periods (Table 5). At an ECV of ≤2 μg/ml, 304 of 310 (98.0%) isolates obtained during 2006–2011 were wild-type for fluconazole as against 417 of 446 (93.4%) isolates tested during 2012–2017 (Table 5). Among 21 fluconazole-resistant *C*. *parapsilosis* isolates, cross-resistance to voriconazole (MIC >1μg/ml) was observed in 7 isolates. One isolate each of *C*. *tropicalis* from both the periods (1 of 112 during 2006–2011 and 1 of 150 during 2012–2017) was resistant to fluconazole. None of the *C*. *dubliniensis* (n = 27), *C*. *orthopsilosis* (n = 7) and *C*. *metapsilosis* (n = 2) isolates were resistant to the four antifungal agents tested including fluconazole (Tables 3 and 4). Although *C*. *glabrata* intrinsically is less susceptible to azoles, a greater number of fluconazole-resistant isolates (14 of 115, 12%) were encountered in 2012–2017 than in 2006–2011 (3 of 74, 4.0%).

**Table 5:** Comparative distribution of four major *Candida* species obtained during 2006–2011 and 2012–2017.

*Candida* species / Antifungal agent	Year of testing	No. of isolates	MIC distribution of isolates (μg/ml by Etest)																							
			≤0.023	0.03	0.047	0.06	0.094	0.125	0.19	0.25	0.38	0.5	0.75	1	1.5	2	3	4	6	8	12	16	24	32	48	≥64
**Amphotericin B**																										
*C*. *albicans*	2006–2011	378	120	65	64	53	31	24	9	5	5		2													
	2012–2017	363	112	50	56	55	51	20	14	3	2															
*C*. *parapsilosis*	2006–2011	310	157	42	42	24	14	13	7	2	4	1	2	2												
	2012–2017	446	229	65	57	40	29	8	7	2	3	3	3													
*C*. *tropicalis*	2006–2011	114	33	10	18	13	14	11	6	4	1	3	1													
	2012–2017	150	21	16	19	20	29	24	13	6	1	1														
*C*. *glabrata*	2006–2011	75	18	4	8	4	4	4	16	10	3	3				**1**										
	2012–2017	115	13	2	11	12	15	22	12	15	5	7												**1**		
**Fluconazole**																										
*C*. *albicans*	2006–2011	371	4	2		7	5	33	49	49	63	48	54	21	21	7	2	3								**3**
	2012–2017	363					2	8	22	37	62	80	65	60	17	2	1		2		**3**	**1**	**1**			
*C*. *parapsilosis*	2006–2011	310	13	3	2	2	7	19	31	52	49	58	34	18	13	3	2	2	1					**1**		
	2012–2017	446			1		10	10	34	35	66	66	86	66	28	15	5	2	1	**2**	**1**		**2**	**2**		**14**
*C*. *tropicalis*	2006–2011	112	3				4	4	5	10	15	10	24	18	6	9	1	1	1	**1**						
	2012–2017	150	1			2		4	16	20	33	31	24	14	3		1									**1**
*C*. *glabrata*	2006–2011	74				1		1	1			1	1		4	1	3	13	6	13	11	4	6	4	1	**3**
	2012–2017	115											1		1	2		22	13	17	13	17	3	9	3	**14**
**Voriconazole**																										
*C*. *albicans*	2006–2011	331	190	31	48	17	18	17	3	2		1		**1**										**3**		
	2012–2017	304	173	24	22	33	15	10	13	3	6		1	**2**	**2**											
*C*. *parapsilosis*	2006–2011	274	209	25	14	7	10	5	2	1	1															
	2012–2017	360	224	48	27	18	7	7	3	5	8	3	3		**1**	**2**	**2**							**2**		
*C*. *tropicalis*	2006–2011	108	34	9	13	10	22	10	5	3	1					**1**										
	2012–2017	117	36	18	13	20	17	6	5	1			1													
*C*. *glabrata*	2006–2011	51	4		1	5	8	6	5	7	5	2	3	1	3			1								
	2012–2017	84		3	2	3	4	6	15	10	19	4	5	2	3	4	1	2						1		
**Caspofungin**																										
*C*. *albicans*	2006–2011	65	14	3	5	10	13	13	2	3	2															
	2012–2017	363	35	18	24	56	77	64	75	13	1															
*C*. *parapsilosis*	2006–2011	32	3			1		4	3	2	5	7	3	3	1											
	2012–2017	446	5	1	3	6	9	11	73	82	154	83	17	2												
*C*. *tropicalis*	2006–2011	29	3		4	6	4	3	3	4	1		1													
	2012–2017	150	8	3	13	9	31	32	43	6	5															
*C*. *glabrata*	2006–2011	9			1	2		2	2	1	1															
	2012–2017	115	8	1	2	12	20	25	39	6	2															

Note: Number of resistant isolates shown in bold.

## Discussion

Invasive fungal infections (IFI) are regarded as the diseases of medical progress. With the changes in clinical practice, the spectrum of fungi capable of causing IFI is also changing, and so is the epidemiology of invasive *Candida* infections. *Candida* infections form a major component of healthcare-associated IFIs and are associated with considerable mortality, varying from 29% to 72% in different patient populations representing different geographic regions/countries [24]. Like-wise, annual incidence of candidemia is also quite variable in different populations and age groups [24]. Surprisingly, the annual incidence rate (number of candidemia cases per 100,000 population) showed a declining trend (from 4.45 to 3.5) in Kuwait over the study period. One likely explanation for this decline is the rapid increase (from 2.377 million in 2006 to 4.136 million in 2017) in the total population of Kuwait particularly during 2010–2014, mostly due to influx of large number of younger (<40 years of age) and healthy expatriate workers who are less likely to have IFIs. In our previous study from Kuwait, we have reported *C*. *albicans* as the predominant species accounting for 39.5% of isolates over a 10-year period (1996–2005), followed by *C*. *parapsilosis* (30.0%), *C*. *tropicalis* (12.5%) and *C*. *glabrat*a (5.6%) [8]. While *C*. *albicans* remained as the most frequently isolated species from candidemia patients during 2006 to 2012, it was replaced by *C*. *parapsilosis* sensu stricto during 2013 to 2016 as the most frequently isolated *Candida* species. Furthermore, the isolation frequency of *C*. *tropicalis* and *C*. *glabrata* also showed an upward trend during the study period. Consistent with these data, the ratio of isolation of *C*. *albicans* versus all six *Candida* species isolates declined nearly consistently from a high of 0.46 in 2006 to a low of 0.33 in 2017. Thus, the proportion of *Candida* species causing candidemia has significantly changed in favor of non-*albicans Candida* species. During the first half of the study (2006–2011), *C*. *albicans* accounted for 41.8% of bloodstream infections, followed by *C*. *parapsilosis* (32.01%), *C*. *tropicalis* (13.59%), and *C*. *glabrata* (8.77%). During the next six-year (2012–2017) period, the proportion of *C*. *albicans* decreased about 9% (33.11%), whereas *C*. *parapsilosis* showed an increase of >5% and the other two species, *C*. *tropicalis* and *C*. *glabrata* of about 2% each.

Our data are consistent with world reports showing that the prevalence of non-*albicans Candida* species is gradually increasing and other *Candida* species such as *C*. *parapsilosis*, *C*. *glabrata* or *C*. *tropicalis* are emerging as more frequent cause of bloodstream infections [1, 25–28]. *C*. *glabrata* has now become the dominant species in some centers of USA, Canada and Europe [4, 6] while *C*. *parapsilosis* has also become the dominant species in some centers/geographical locations [4, 28–30]. Additionally, many less common and newly recognized *Candida* species including *Candida auris* are being isolated with increasing frequency [31–35]

A comparative distribution of bloodstream isolates of *Candida* species from the Middle East is presented in Table 6 [8, 12, 36–47]. Recent studies from Qatar [43], Israel [44] and Iran [45] also indicate predominance of non-*albicans Candida* species as compared to *C*. *albicans*. Generally, the prevalence of *C*. *tropicalis* and *C*. *glabrata* has been reported to be ≤20% with one exception from Israel, where a higher rate of *C*. *glabrata* (23.7%) was reported in adult patients (Table 6) [8, 12, 36–61]. This shift towards non-*albicans Candida* species is consistent with many previous reports [1, 6, 24]. However, this shift is also somewhat species-specific. The largest proportional increase in *C*. *glabrata* has been observed in USA [4, 29], Australia [48] and Belgium [49]. In contrast, several surveillance and multicenter studies in other countries, such as Spain [50], Latin America [26, 28] and South Africa [51] have indicated the emergence of *C*. *parapsilosis* as the second most important cause of bloodstream infection after *C*. *albicans*. Consistent with many other studies from North America, Latin America, China and Europe [26, 28, 29, 52], *C*. *tropicalis* is the third most common cause of bloodstream infection in Kuwait. This is in contrast to countries of Asian Pacific region [53] as well as India [54] and Pakistan [55] where *C*. *tropicalis* is the second most common bloodstream pathogen after *C*. *albicans*. The epidemiologic factors that might have contributed to predominance of *C*. *tropicalis* in these countries are not fully understood.

**Table 6:** Distribution of *Candida* species isolated from candidaemia patients in different studies reported from the Middle East.

Reference	Study period	Country	No. of isolates	% Distribution of Candida species						% Ratio *C. albicans*/ non *albicans*
				*C*. *albicans*	*C*. *parapsilosis*	*C*. *tropicalis*	*C*. *glabrata*	*C*. *krusei*	Others	
Rennert *et al*. [36]	1994	Israel	298	53.6	11.9	10.9	6.5	0.7	15.9	53.6/45.9
Bukharie [37]	1995–2000	Saudi Arabia	32	19	44	25	3	6	3	19/81
Ellis *et al*. [38]	1995–2001	UAE	60	45	5	15	5	0	30	45/55
Al-Jasser & Elkhizzi [39]	1996–2002	Saudi Arabia	294	50.7	10.9	20.7	7.1	7.8	3.1	50.7/49.6
Al-Sweih *et al*. [12]	1997–2006	Kuwait	153	41.2	49	-	1.3	-	8.49	41.2/58.79
Osoba *et al*. [40]	1998–2002	Saudi Arabia	83	46	10.8	10.8	4.8	6	21.6	46/54
Al-Essa *et al*. [41]	1997	Kuwait	22	31.8	13.7	-	-	-	54.5	31.8/68.2
Mokaddas *et al*. [42]	1994–1998	Kuwait	25	56	28	8	8	-	10.8	56/54.8
Mokaddas *et al*. [8]	1996–2005	Kuwait	607	39.5	30.6	12.4	5.6	1.6	11.8	39.5/62
Taj-Aldeen *et al*. [43]	2004–2010	Qatar	201	33.8	20.8*	17.9	18.9	-	8.5	33.8/45.3
Eliakim-raz *et al*. [44]	2007–2014	Israel	118**	44	15.2	13.5	23.7	0.8	2.54	44/55.74
Vaezi *et al*. [45]	until 2015	Iran	55	27.2	30.9	14.5	18.1	-	9	27.2/72.5
Obaid & Khan [46]	2014–2016	Kuwait	82	32	32	20	13	-	3.65	32/68.65
Taj-Aldeen *et al*. [47]	2009–2014	Qatar	301	30.2	17.9	17.9	25.5	-	8.3	30.2/69.6
Present study	2006–2011	Kuwait	970	41.75	34.2	12.98	7.8	1.54	-	41.75/56.52
	2012–2017		1105	32.8	40.72	13.57	10.4	1.44	-	32.8/66.13

*Includes 8 isolates of *C*. *orthopsilosis;*

**Includes population of >18 years; United Arab Emirates (UAE)

Our data of antifungal susceptibility suggest that resistance is still uncommon (<5%) but it is beginning to emerge against fluconazole particularly in *C*. *albicans* and *C*. *parapsilosis*. The comparison of MIC data clearly demonstrates that there is a noticeable increase in fluconazole MICs of *C*. *albicans* as well as of *C*. *parapsilosis* among the isolates obtained during 2012–2017 as compared to 2006–2011. The first fluconazole-resistant *C*. *albicans* isolate from blood was detected in March 2011. Subsequently, the number of resistant isolates recorded during 2006–2011 and 2012–2017 increased to three (0.8%) and five (1.37%), respectively. The reduction in fluconazole susceptibility was also reflected by MIC distribution. At an ECV of ≤0.5 μg/ml, 260 of 371 (70.1%) *C*. *albicans* isolates obtained during 2006–2011 were wild-type for fluconazole as compared to 211 of 363 (58.1%) isolates during 2012–2017. Emergence of fluconazole resistance was more conspicuous and statistically significant among *C*. *parapsilosis* isolates, where 21 (4.7%) of the isolates were found to be resistant to this drug during 2012–2017. This trend was also supported by the fact that at an ECV of ≤2 μg/ml, 304 of 310 (98.0%) isolates obtained during 2006–2011 were wild-type for fluconazole as compared to 93.4% (417 of 446) during 2012–2017 (Table 5). Notably, none of our fluconazole-resistant isolate came from a neonate. The first fluconazole-resistant bloodstream isolate of *C*. *parapsilosis* was detected in April 2010 and gradually resistant strains have spread to other hospitals. In a study of 442 *C*. *parapsilosis* sensu stricto isolates, 5 of 11 (45%) fluconazole-resistant isolates had Y132F mutation in *ERG*11 and originated from a single hospital [56]. Four of these isolates came from blood and one from sputum. It is not known if these patients had fluconazole exposure prior to developing resistance. Since two of the patients were females, it is possible that they might have used fluconazole for vaginal symptoms. In a previous study, Grossman et al. (2015) [57] reported two mechanisms that contribute to fluconazole resistance in *C*. *parapsilosis*, that is by an amino acid substitution in *ERG*11 gene and by overexpression of the efflux pump MDR1, possibly due to point mutations in the MRR1 transcription factor that regulates MDR1. Although there is no clear evidence that prior exposure to fluconazole can be linked to emergence of resistance [51, 58], nonetheless, reports of outbreak of candidemia caused by resistant strain following prolonged antifungal therapy have begun to emerge [59, 60], thus limiting treatment of choice to amphotericin B or an echinocandin, although the latter drug is relatively less active but still therapeutically used against *C*. *parapsilosis* candidemia. Our data seem to suggest that *C*. *parapsilosis* may have a greater propensity to develop acquired resistance to azoles than *C*. *albicans* or *C*. *tropicalis*. The report highlights the need of strict compliance of hand sepsis protocols to prevent transmission of fluconazole-resistant strains of *C*. *parapsilosis* through healthcare staff. It is worth mentioning that in a recent study, 11 (52%) of 21 fluconazole-resistant bloodstream isolates came from a single hospital and belonged to a single clone (unpublished data).

In most of the published studies resistance to fluconazole has been <10% with few exceptions [24]. In a study from Spain, a high rate of fluconazole resistance (27.6%) has been observed among *Candida* isolates obtained from hematology and oncology patients [50]. Similarly, among *C*. *parapsilosis* isolates from South Africa, only 37% were found susceptible to fluconazole and voriconazole and 44% of fluconazole-resistant isolates also showed cross-resistance to voriconazole. Unlike higher rates of fluconazole resistance among *C*. *tropicalis* isolates in China [62, 63] and Australia [48], it was uncommon/rare (0.38%) in Kuwait. In India, also where *C*. *tropicalis* has emerged as the most common bloodstream pathogen (n = 382; 41.6%), resistance to fluconazole was uncommon (2.6%) and same is true in the Asia Pacific region [63]. *C*. *glabrata* is known to be intrinsically less susceptible to fluconazole, however, there was an indication that this species is also becoming less susceptible to this drug. Of 74 *C*. *glabrata* isolates tested during 2006–2011, 10.8% isolates were inhibited at MICs of 32 μg/ml or more as compared to 22.6% (n = 115) tested during 2012–2017 and resistance to fluconazole was restricted to only 12%. In Kuwait, caspofungin is in clinical use for several years. However, none of the *Candida* species isolates developed resistance to caspofungin, so far.

Our study has two major limitations, firstly, because of retrospective nature, clinical details of the patients including antifungal treatment received are not available, and secondly, it is possible that not all *Candida* species isolates from candidemia cases were referred for identification and antifungal susceptibility testing to MRL by different hospitals.

In conclusion, our data indicate that during the two study periods there is a shift of 8.8% in favor of non-*albicans Candida* species causing candidemia in Kuwait. Additionally, our antifungal susceptibility data suggest a gradual decrease in susceptibility to fluconazole, particularly among *C*. *albicans* and *C*. *parapsilosis*, which may be a prelude of emergence of greater number of resistant strains in future. While resistance to fluconazole and other antifungal agents is still uncommon, it calls for a continued need of surveillance and strengthening of antifungal stewardship policies to minimize acquisition of acquired resistance.

## References

[1] Guinea J. Global trends in the distribution of *Candida* species causing candidemia. Clin Microbiol Infect. 2014;20 Suppl 6:5–10. 10.1111/1469-0691.12539 24506442

[2] Wu PF, Liu WL, Hsieh MH, Hii IM, Lee YL, Lin YT, Ho MW, Liu CE, Chen YH, Wang FD. Epidemiology and antifungal susceptibility of candidemia isolates of non-*albicans Candida* species from cancer patients. Emerg Microbes Infect. 2017;6(10):e87 10.1038/emi.2017.74 29018251 PMC5658770

[3] McCarty TP, Pappas PG. Invasive candidiasis. Infect Dis Clin North Am. 2016; 30(1):103–24. 10.1016/j.idc.2015.10.013 26739610

[4] Pfaller MA, Andes DR, Diekema DJ et al Epidemiology and outcomes of invasive candidiasis due to non-albicans species of *Candida* in 2,496 patients: data from the prospective antifungal therapy (PATH) registry 2004–2008. PLoS One 2014; 9: e101510. 10.1371/journal.pone.0101510 PMC4081561 24991967

[5] Quindós G. Epidemiology of candidaemia and invasive candidiasis. A changing face. Rev Iberoam Micol. 2014; 31:42–48. 10.1016/j.riam.2013.10.001 24270071

[6] Astvad KMT, Johansen HK, Røder BL, Rosenvinge FS, Knudsen JD, Lemming L, Schønheyder HC, Hare RK, Kristensen L, Nielsen L, Gertsen JB, Dzajic E, Pedersen M, Østergård C, Olesen B, Søndergaard TS, Arendrup MC. Update from a 12-Year Nationwide fungemia surveillance: increasing intrinsic and acquired resistance causes concern. J Clin Microbiol. 2018;56. pii: e01564-17. 10.1128/JCM.01564-17 PMC5869841 29212705

[7] Tan BH, Chakrabarti A, Li RY, Patel AK, Watcharananan SP, Liu Z, Chindamporn A, Tan AL, Sun PL, Wu UI, Chen YC; Asia Fungal Working Group (AFWG). Incidence and species distribution of candidaemia in Asia: a laboratory-based surveillance study. Clin Microbiol Infect. 2015;21: 946–53. 10.1016/j.cmi.2015.06.010 26100373

[8] Mokaddas EM, Al-Sweih NA, Khan ZU. Species distribution and antifungal susceptibility of *Candida* bloodstream isolates in Kuwait: a 10-year study. J Med Microbiol. 2007;56(Pt 2):255–259. 10.1099/jmm.0.46817-0 17244809

[9] Khan ZU, Al-Sweih NA, Ahmad S, Al-Kazemi N, Khan S, Joseph L, Chandy R. Outbreak of fungemia among neonates caused by *Candida haemulonii* resistant to amphotericin B, itraconazole, and fluconazole. J Clin Microbiol. 2007;45: 2025–2027. 10.1128/JCM.00222-07 17428940 PMC1933024

[10] Khan Z, Ahmad S, Joseph L, Chandy R. *Candida dubliniensis*: an appraisal of its clinical significance as a bloodstream pathogen. PLoS One. 2012;7: e32952 10.1371/journal.pone.0032952 22396802 PMC3292580

[11] Al-Haqqan A, Al-Sweih N, Ahmad S, Khan S, Joseph L, Varghese S, Khan Z. Azole-resistant *Candida blankii* as a newly recognized cause of bloodstream infection. New Microbes New Infect. 2018; 26:25–29. 10.1016/j.nmni.2018.06.008 30245830 PMC6141729

[12] Al-Sweih N, Khan Z, Khan S, Devarajan LV. Neonatal candidaemia in Kuwait: a 12-year study of risk factors, species spectrum and antifungal susceptibility. Mycoses. 2009;52(6):518–23. 10.1111/j.1439-0507.2008.01637.x 18983425

[13] Al-Sweih N, Ahmad S, Joseph L, Khan S, Vayalil S, Chandy R, Khan Z. *Candida fermentati* as a cause of persistent fungemia in a preterm neonate successfully treated by combination therapy with amphotericin B and caspofungin. J Clin Microbiol. 2015; 53:1038–41. 10.1128/JCM.03351-14 25568433 PMC4390630

[14] Al-Sweih N, Ahmad S, Khan S, Khan Z, Joseph L, Vayalil S, Chandy R. Persistent *Candida conglobata* bloodstream infection in a preterm neonate successfully treated by combination therapy with amphotericin B and caspofungin. J Mycol Med. 2017;27:271–276. 10.1016/j.mycmed.2017.01.010 28189376

[15] Al-Sweih N, Ahmad S, Khan S, Joseph L, Asadzadeh M, Khan Z. *Cyberlindnera fabianii* fungaemia outbreak in preterm neonates in Kuwait and literature review. Mycoses. 2019; 62:51–61. 10.1111/myc.12846 30184277

[16] Ahmad S, Khan Z, Mustafa AS, Khan ZU. Seminested PCR for diagnosis of candidemia: comparison with culture, antigen detection, and biochemical methods for species identification. J Clin Microbiol 2002; 40: 2483–2489. 10.1128/JCM.40.7.2483-2489.2002 12089267 PMC120535

[17] Khan ZU, Ahmad S, Hagen F, Fell JW, Kowshik T, Chandy R, Boekhout T. *Cryptococcus randhawai* sp. nov., a novel anamorphic basidiomycetous yeast isolated from tree trunk hollow of *Ficus religiosa* (peepal tree) from New Delhi, India. Antonie Van Leeuwenhoek 2010; 97: 253–259. 10.1007/s10482-009-9406-8 20091225

[18] Asadzadeh M, Ahmad S, Al-Sweih N, Khan ZU Rapid molecular differentiation and genotypic heterogeneity among *Candida parapsilosis* and *Candida orthopsilosis* strains isolated from clinical specimens in Kuwait. J Med Microbiol. 2009;58 (Pt 6):745–52. 10.1099/jmm.0.008235-0 19429750

[19] Asadzadeh M, Ahmad S, Al-Sweih N, Gulati RR, Khan Z. First isolation of *Candida metapsilosis* in Kuwait, an emerging global opportunistic pathogen. J Mycol Med. 2016;26:46–50. 10.1016/j.mycmed.2015.11.001 26700651

[20] Jamal WY, Ahmad S, Khan ZU, Rotimi VO. Comparative evaluation of two matrix-assisted laser desorption/ionization time-of-flight mass spectrometry (MALDI-TOF MS) systems for the identification of clinically significant yeasts. Int J Infect Dis. 2014 9;26:167–70. 10.1016/j.ijid.2014.05.031 25080355

[21] CLSI. *Performance Standards for Antifungal*. *Susceptibility Testing of Yeasts*. 1^st^ Edition, CLSI supplement M60, Wayne, PA: Clinical and Laboratory Standards Institute 2017.

[22] Lass-Flörl C, Arendrup MC, Rodriguez-Tudela JL, Cuenca-Estrella M, Donnelly P, Hope W; European Committee on Antimicrobial Susceptibility Testing-Subcommittee on Antifungal Susceptibility Testing. EUCAST technical note on Amphotericin B. Clin Microbiol Infect. 2011;17(12): E27–29. 10.1111/j.1469-0691.2011.03644.x 22011310

[23] Pfaller MA, Diekema DJ. Progress in antifungal susceptibility testing of *Candid*a spp. by use of Clinical and Laboratory Standards Institute broth microdilution methods, 2010 to 2012. J Clin Microbiol. 2012;50(9):2846–2856 10.1128/JCM.00937-12 22740712 PMC3421803

[24] Lamoth F, Lockhart SR, Berkow EL, Calandra T. Changes in the epidemiological landscape of invasive candidiasis. J Antimichrob Chemother 2018;73 (suppl 1):i4–i13. 10.1093/jac/dkx444 PMC11931512 29304207

[25] Cleveland AA, Harrison LH, Farley MM et al Declining incidence of candidemia and the shifting epidemiology of *Candida* resistance in two US metropolitan areas, 2008–2013: results from a population-based surveillance. PLoS One 2015; 10: e0120452. 10.1371/journal.pone.0120452 PMC4378850 25822249

[26] Doi AM, Pignatari AC, Edmond MB, Marra AR, Camargo LF, Siqueira RA, da Mota VP, Colombo AL. Epidemiology and microbiologic characterization of nosocomial candidemia from a Brazilian National Surveillance Program. PLoS One 2016; 11: e0146909 10.1371/journal.pone.0146909 26808778 PMC4726651

[27] Rodriguez L, Bustamante B, Huaroto L, Agurto C, Illescas R, Ramirez R, Diaz A, Hidalgo J. A multi-centric Study of *Candida* bloodstream infection in Lima-Callao, Peru: Species distribution, antifungal resistance and clinical outcomes. PLoS One. 2017;12: e0175172 10.1371/journal.pone.0175172 28419092 PMC5395148

[28] Nucci M, Queiroz-Telles F, Alvarado-Matute T Tiraboschi IN, Cortes J, Zurita J, Guzman-Blanco M, Santolaya ME, Thompson L, Sifuentes-Osornio J, Echevarria JI, Colombo AL; Latin American Invasive Mycosis Network. Epidemiology of candidemia in Latin America: a laboratory-based study. PLoS One 2013; 8: e59373 10.1371/journal.pone.0059373 23527176 PMC3601956

[29] Lockhart SR, Iqbal N, Ahlquist AM et al Species identification and antifungal susceptibility of *Candida* bloodstream isolates from population-based surveillance in two US cities: 2008–2011. J Clin Microbiol 2012; 50: 3435–3442. 10.1128/JCM.01283-12 22875889 PMC3486211

[30] Delfino D. et al. Potential association of specific *Candida parapsilosis* genotypes, bloodstream infections and colonization of health workers’ hands. *Clin*. *Microbiol*. *Infect*. 20, 2014, O946–O951. 10.1111/1469-0691.12685 24845557

[31] Chen S, Slavin M, Nguyen Q, Marriott D, Playford EG, Ellis D, Sorrell T; Australian candidemia study. Active surveillance for candidaemia, Australia. Emerg Infect Dis 2006; 12: 1508–1516. 10.3201/eid1210.060389 17176564 PMC3290948

[32] Jung DS, Farmakiotis D, Jiang Y, Tarrand JJ, Kontoyiannis DP. Uncommon *Candida* Species Fungemia among Cancer Patients, Houston, Texas, USA. Emerg Infect Dis. 2015;21(11):1942–50. 10.3201/eid2111.150404 26488845 PMC4625381

[33] Tsai MH, Hsu JF, Yang LY, Pan YB, Lai MY, Chu SM, Huang HR, Chiang MC, Fu RH, Lu JJ. Candidemia due to uncommon *Candida* species in children: new threat and impacts on outcomes. Sci Rep. 2018 10 15;8(1):15239 10.1038/s41598-018-33662-x 30323257 PMC6189077

[34] Jeffery-Smith A, Taori SK, Schelenz S, Jeffery K, Johnson EM, Borman A, Candida auris Incident Management Team, Manuel R, Brown CS. *Candida auris*: a review of the Literature. Clin Microbiol Rev. 2017;31. pii: e00029-17. 10.1128/CMR.00029-17 PMC5740969 29142078

[35] Khan Z, Ahmad S, Al-Sweih N, Joseph L, Alfouzan W, Asadzadeh M. Increasing prevalence, molecular characterization and antifungal drug susceptibility of serial *Candida auris* isolates in Kuwait. PLoS One 2018; 13: e0195743 10.1371/journal.pone.0195743 29630658 PMC5891028

[36] Rennert G, Rennert HS, Pitlik S, Finkelstein R, Kitzes-Cohen R. Epidemiology of candidemia—a nationwide survey in Israel. Infection. 2000;28:26–29. 10697787 10.1007/s150100050006

[37] Bukharie HA. Nosocomial candidemia in a tertiary care hospital in Saudi Arabia. Mycopathologia. 2002;153:195–198. 12014479 10.1023/a:1014945517790

[38] Ellis M, Hedstrom U, Jumaa P, Bener A. Epidemiology, presentation, management and outcome of candidemia in a tertiary care teaching hospital in the United Arab Emirates, 1995-2001.Med Mycol. 2003;41:521–528. 14725327 10.1080/13693780310001645337

[39] Al-Jasser AM, Elkhizzi NA. Distribution of *Candida* species among bloodstream isolates. Saudi Med J. 2004;25:566–569. 15138521

[40] Osoba AO, Al-Mowallad AW, McAlear DE, Hussein BA. Candidemia and the susceptibility pattern of *Candida* isolates in blood. Saudi Med J. 2003;24:1060–1063. 14578968

[41] Al-Essa M. Khan Z. Rashwan N. Kazi A. Pattern of candidiasis in the newborn: A Study from Kuwait. Med Principles Pract 2000; 9:174–180.

[42] Mokaddas EM, Ramadan SA, Abo el Maaty SH, Sanyal SC. Candidemia in pediatric surgery patients. J Chemother. 2000; 12:332–338. 10.1179/joc.2000.12.4.332 10949983

[43] Taj-Aldeen SJ, Kolecka A, Boesten R, Alolaqi A, Almaslamani M, Chandra P, Meis JF, Boekhout T. Epidemiology of candidemia in Qatar, the Middle East: performance of MALDI-TOF MS for the identification of *Candida* species, species distribution, outcome, and susceptibility pattern. Infection. 2014; 42:393–404 10.1007/s15010-013-0570-4 24352810

[44] Eliakim-Raz N, Babaoff R, Yahav D, Yanai S, Shaked H, Bishara J. Epidemiology, microbiology, clinical characteristics, and outcomes of candidemia in internal medicine wards-a retrospective study. Int J Infect Dis. 2016;52:49–54. 10.1016/j.ijid.2016.09.018 27663909

[45] Vaezi A, Fakhim H, Khodavaisy S, Alizadeh A, Nazeri M, Soleimani A, Boekhout T, Badali H. Epidemiological and mycological characteristics of candidemia in Iran: A systematic review and meta-analysis. J Mycol Med. 2017;27:146–152. 10.1016/j.mycmed.2017.02.007 28318900

[46] Alobaid K Khan Z. Epidemiologic characteristics of adult candidemic patients in a secondary hospital in Kuwait: A retrospective study. J Mycol Med. 2018 pii: S1156-5233(18)30093-3. 10.1016/j.mycmed.2018.12.001 [Epub ahead of print] 30578148

[47] Taj-Aldeen SJ, Salah H, Perez WB, Almaslamani M, Motyl M, AbdulWahab A, Healey KR, Perlin DS. Molecular Analysis of Resistance and Detection of Non-Wild-Type Strains Using Etest Epidemiological Cutoff Values for Amphotericin B and Echinocandins for Bloodstream *Candida* Infections from a Tertiary Hospital in Qatar. Antimicrob Agents Chemother. 2018 8 27;62(9). pii: e00214–18 10.1128/AAC.00214-18 29941644 PMC6125493

[48] Chapman B, Slavin M, Marriott D, Halliday C, Kidd S, Arthur I, Bak N, Heath CH, Kennedy K, Morrissey CO, Sorrell TC, van Hal S, Keighley C, Goeman E, Underwood N, Hajkowicz K, Hofmeyr A, Leung M, Macesic N, Botes J, Blyth C, Cooley L, George CR, Kalukottege P, Kesson A, McMullan B, Baird R, Robson J, Korman TM, Pendle S, Weeks K, Liu E, Cheong E, Chen S; Australian and New Zealand Mycoses Interest Group. Changing epidemiology of candidaemia in Australia. J Antimicrob Chemother. 2017;72: 1103–1108. 10.1093/jac/dkw422 28364558

[49] Trouvé C, Blot S, Hayette MP, Jonckheere S, Patteet S, Rodriguez-Villalobos H, Symoens F, Van Wijngaerden E, Lagrou K. Epidemiology and reporting of candidaemia in Belgium: a multi-centre study. Eur J Clin Microbiol Infect Dis. 2017 4;36(4):649–655. 10.1007/s10096-016-2841-3 27858242

[50] Puig-Asensio M, Padilla B, Garnacho-Montero J, Zaragoza O, Aguado JM, Zaragoza R, Montejo M, Muñoz P, Ruiz-Camps I, Cuenca-Estrella M, Almirante B; CANDIPOP Project; GEIH-GEMICOMED (SEIMC); REIPI. Epidemiology and predictive factors for early and latemortality in *Candida* bloodstream infections: a population-based surveillance in Spain. Clin Microbiol Infect 2014; 20: O245–254. 10.1111/1469-0691.12380 24125548

[51] Govender NP, Patel J, Magobo RE, Naicker S, Wadula J, Whitelaw A, Coovadia Y, Kularatne R, Govind C, Lockhart SR, Zietsman IL; TRAC-South Africa group. Emergence of azole-resistant *Candida parapsilosis* causing bloodstream infection: results from laboratory-based sentinel surveillance in South Africa. J Antimicrob Chemother. 2016;71(7):1994–2004. 10.1093/jac/dkw091 27125552 PMC11911919

[52] Xiao M, Sun ZY, Kang M, Guo DW, Liao K, Chen SC, Kong F, Fan X, Cheng JW, Hou X, Zhou ML, Li Y, Yu SY, Huang JJ, Wang H, Xu YC; China Hospital Invasive Fungal Surveillance Net (CHIF-NET) Study Group. Five-Year National Surveillance of Invasive Candidiasis: Species Distribution and Azole Susceptibility from the China Hospital Invasive Fungal Surveillance Net (CHIF-NET) Study. J Clin Microbiol. 2018; 56. pii: e00577-18. 10.1128/JCM.00577-18 PMC6018329 29743305

[53] Tan TY, Hsu LY, Alejandria MM, Chaiwarith R, Chinniah T, Chayakulkeeree M, Choudhury S, Chen YH, Shin JH, Kiratisin P, Mendoza M, Prabhu K, Supparatpinyo K, Tan AL, Phan XT, Tran TT, Nguyen GB, Doan MP, Huynh VA, Nguyen SM, Tran TB, Van Pham H. Antifungal susceptibility of invasive *Candida* bloodstream isolates from the Asia-Pacific region.Med Mycol. 2016;54:471–477. 10.1093/mmy/myv114 26868904

[54] Chakrabarti A, Sood P, Rudramurthy SM, Chen S, Kaur H, Capoor M, Chhina D, Rao R, Eshwara VK, Xess I, Kindo AJ, Umabala P, Savio J, Patel A, Ray U, Mohan S, Iyer R, Chander J, Arora A, Sardana R, Roy I, Appalaraju B, Sharma A, Shetty A, Khanna N, Marak R, Biswas S, Das S, Harish BN, Joshi S, Mendiratta D. Incidence, characteristics and outcome of ICU-acquired candidemia in India. Intensive Care Med. 2015: 10.1007/s00134-014-3603-2 Epub 2014 Dec 16. 25510301

[55] Farooqi JQ, Jabeen K, Saeed N, Iqbal N, Malik B, Lockhart SR, Zafar A, Brandt ME, Hasan R. Invasive candidiasis in Pakistan: clinical characteristics, species distribution and antifungal susceptibility. J Med Microbiol 2013; 62: 259–68. 10.1099/jmm.0.048785-0 23105021 PMC4629241

[56] Asadzadeh M, Ahmad S, Al-Sweih N, Khan Z. Epidemiology and molecular basis of resistance to fluconazole among clinical *Candida parapsilosis* Isolates in Kuwait. Microb Drug Resist. 2017; 23:966–972. 10.1089/mdr.2016.0336 28353392

[57] Grossman NT, Pham CD, Cleveland AA, Lockhart SR.Molecular mechanisms of fluconazole resistance in *Candida parapsilosis* isolates from a U.S. surveillance system. Antimicrob Agents Chemother. 2015;59:1030–1037. 10.1128/AAC.04613-14 25451046 PMC4335858

[58] Naicker SD, Govender N, Patel J, Zietsman IL, Wadula J, Coovadia Y, Kularatne R, Seetharam S, Govender NP; TRAC-SA group. Comparison of species-level identification and antifungal susceptibility results from diagnostic and reference laboratories for bloodstream *Candida* surveillance isolates, South Africa, 2009–2010. Med Mycol. 2016;54:816–824 10.1093/mmy/myw046 27335055

[59] Zhang L, Xiao M, Watts MR, Wang H, Fan X, Kong F, Xu YC. Development of fluconazole resistance in a series of *Candida parapsilosis i*solates from a persistent candidemia patient with prolonged antifungal therapy. BMC Infect Dis. 2015;15:340 10.1186/s12879-015-1086-6 26282840 PMC4539859

[60] Pinhati HM, Casulari LA, Souza AC, Siqueira RA, Damasceno CM, Colombo AL. Outbreak of candidemia caused by fluconazole resistant *Candida parapsilosis* strains in an intensive care unit. BMC Infect Dis. 2016 8 20;16(1):433 10.1186/s12879-016-1767-9 27544427 PMC4992558

[61] Guo LN, Xiao M, Cao B, Qu F, Zhan YL, Hu YJ, Wang XR, Liang GW, Gu HT, Qi J, Yuan H, Min R, Wang FY, Liu LJ, Wang HB, Jiang W, Duan XG, Xu WJ, Yu YH, Su JR, Zhang JZ, Nong JQ, Liu SM, Li J, Liu JT, Yue ZG, Yang D, Guo J, Zhao R, Zhang YN, Yang XM, Liu XQ, Hsueh PR, Xu YC. Epidemiology and antifungal susceptibilities of yeast isolates causing invasive infections across urban Beijing, China. Future Microbiol. 2017;12: 1075–1086. 10.2217/fmb-2017-0036 28836465

[62] Fan X, Xiao M, Zhang D, Huang JJ, Wang H, Hou X, Zhang L, Kong F, Chen SC, Tong ZH, Xu Molecular mechanisms of azole resistance in *Candida tropicalis* isolates causing invasive candidiasis in China. Clin Microbiol Infect. 2018 11 22. pii: S1198-743X (18)30734-1. 10.1016/j.cmi.2018.11.007 30472420

[63] Wang H, Xu YC, Hsueh PR. Epidemiology of candidemia and antifungal susceptibility in invasive *Candida* species in the Asia-Pacific region. Future Microbiol. 2016;11:1461–1477. 10.2217/fmb-2016-0099 27750452

